# Inequalities for Jensen–Sharma–Mittal and Jeffreys–Sharma–Mittal Type *f*–Divergences

**DOI:** 10.3390/e23121688

**Published:** 2021-12-16

**Authors:** Paweł A. Kluza

**Affiliations:** Department of Applied Mathematics and Computer Science, University of Life Sciences in Lublin, 28 Głęboka Street, 20-612 Lublin, Poland; pawel.kluza@up.lublin.pl; Tel.: +48-81-531-96-19

**Keywords:** convex function, Csiszár *f*-divergence, Sharma–Mittal *f*-divergence, Jensen–Sharma–Mittal divergence, Jeffreys–Sharma–Mittal divergence, 94A17, 26D15, 15B51

## Abstract

In this paper, we introduce new divergences called Jensen–Sharma–Mittal and Jeffreys–Sharma–Mittal in relation to convex functions. Some theorems, which give the lower and upper bounds for two new introduced divergences, are provided. The obtained results imply some new inequalities corresponding to known divergences. Some examples, which show that these are the generalizations of Rényi, Tsallis, and Kullback–Leibler types of divergences, are provided in order to show a few applications of new divergences.

## 1. Introduction

The Sharma–Mittal entropy was introduced as a new measure of information with two parameters [1]. It has previously been studied in the context of multi-dimensional harmonic oscillator systems [2]. This entropy could also be formulated in the form of exponential families, to which many usual statistical distributions including the Gaussians and discrete multinomials (that is, normalized histograms) belong. In physical applications it plays a major role in the field of thermo-statistics [3].

The Sharma–Mittal entropy is also applied for the analysis of the results of machine learning methods [4,5]. Additionally, the divergence based on considered entropy could be a cost function in the context of so-called the Twin Gaussian Processes [6].

It was originally showed by [7] that the Sharma–Mittal entropy generalized both Tsallis and Rényi entropy in the limiting cases of these two entropies. In [8], authors suggested a physical meaning of Sharma–Mittal entropy, which is the free energy difference between the equilibrium and the off-equilibrium distribution.

Recently, was published a manuscript showing, in opposition to the work [8], that Sharma–Mittal entropy besides the convenient thermodynamic systems does not reduce only to Kullback–Leibler entropy. In [9] Verma and Merigó present the use of Sharma–Mittal entropy under intuitionistic fuzzy environment. Additionally, in [5] Koltcov et al. demonstrate that Sharma–Mittal entropy is a tool for selecting both the number of topics and the values of hyper-parameters, simultaneously controlling for semantic stability, which none of the existing metrics can do.

Another applications of considered entropy are interesting results in the cosmological setup, such as black hole thermodynamics [10]. Namely, it helps us to describe the current accelerated universe by using the vacuum energy in a suitable manner [11]. In addition [12] have established the relation between anomalous diffusion process and Sharma–Mittal entropy.

This paper is based on publications in which we introduced new types of *f*-divergences [13,14,15,16].

In this paper we generalize Sharma–Mittal types divergences in order to obtain new types of divergences and hence the inequalities from which it will be possible to derive new results and generalizations for known divergences in order to estimate the lower and upper bounds which determine the level of the uncertainty measure.

## 2. Sharma–Mittal Type Divergences

Throughout $R_{+}$ and $R_{++}$ denote the sets of non-negative and positive numbers, respectively, i.e., $R_{+}=\left[0,\infty \right)$ and $R_{++}=\left(0,\infty \right)$.

Let $p=(p_{1},\ldots ,p_{n})$ and $q=(q_{1},\ldots ,q_{n})$ with $p_{i},q_{i}\geq 0$, i=1,…,n. The *relative entropy* (also called *Kullback–Leibler divergence*) is defined by (see [17])

(1) $$H_{1}\left(p,q\right)=\sum_{i=1}^{n}p_{i}\log\frac{p_{i}}{q_{i}}.$$

In the above definition, based on continuity arguments, we use a convention that 0log(0/q)=0 and plog(p/0)=+∞. Additionally 0log(0/0)=0.

Let *f*: $R_{+}\to R$ be a convex function on $R_{+}$, and $p=\left(p_{1},\ldots ,p_{n}\right)\in R_{++}^{n}$, $q=\left(q_{1},\ldots ,q_{n}\right)\in R_{+}^{n}$.

The *Csiszár f-divergence* is defined by (see [15])

(2) $$C_{f}\left(p,q\right)=\sum_{i=1}^{n}p_{i}f\left(\frac{q_{i}}{p_{i}}\right).$$

with the conventions $0f\left(\frac{0}{0}\right)=0$ and $0f\left(\frac{c}{0}\right)=c\lim_{t\to \infty }\frac{f(t)}{t}$, c>0 (see [18,19,20]).

The *Tsallis* divergence of order α is defined by (see [17])

$$T_{\alpha }\left(p,q\right)=\frac{1}{\alpha -1}\left(\sum_{i=1}^{n}p_{i}^{\alpha }q_{i}^{1-\alpha }-1\right).$$

The *Rényi* divergence of order α is defined by (see [17,21])

$$H_{\alpha }\left(p,q\right)=\frac{1}{\alpha -1}\log\sum_{i=1}^{n}p_{i}^{\alpha }q_{i}^{1-\alpha }.$$

The *Sharma–Mittal* divergence of order α and degree β is defined by (see [4])

(3) $$SM_{\alpha ,\beta }\left(p,q\right)=\frac{1}{\beta -1}\left[{\left(\sum_{i=1}^{n}p_{i}^{\alpha }q_{i}^{1-\alpha }\right)}^{\frac{1-\beta }{1-\alpha }}-1\right],$$

for all α>0, α≠1 and β≠1.

Let g:I→R be a convex function on an interval I⊂R. Let $x=\left(x_{1},\ldots ,x_{n}\right)\in I^{n}$ and $p_{i}\in \left[0,1\right)$ for i=1,…n.

The *Jensen’s inequality* is as follows (see [22])

(4) $$g\left(\sum_{i=1}^{n}p_{i}x_{i}\right)\leq \sum_{i=1}^{n}p_{i}g\left(x_{i}\right).$$

When the function $k:R_{+}\to R$ is convex and the function $l:R_{+}\to R$ is convex and increasing then the composition of the functions $k\circ l:R_{+}\to R$ is convex. We assume that the probabilities $p_{i}\geq 0$ and $q_{i}>0$ for i=1,…,n.

It is known (see [4]) that if

$$\alpha =\beta \to 1\;\mathrm{then}\;SM_{\alpha ,\beta }\left(p,q\right)\to H_{1}\left(p,q\right),$$

$$\beta \to 1\;\mathrm{and}\;\alpha \in R\;\mathrm{then}\;SM_{\alpha ,\beta }\left(p,q\right)\to H_{\alpha }\left(p,q\right),$$

$$\beta =\alpha \;\mathrm{then}\;SM_{\alpha ,\beta }\left(p,q\right)\to T_{\alpha }\left(p,q\right).$$

Let h:R→R be the differentiable function. Then the *Sharma–Mittal h-divergence* is defined as follows:

(5) $$SM_{h,\alpha ,\beta }\left(p,q\right)=\frac{h\left({\left[\overset{n}{\underset{i=1}{\sum }}p_{i}^{\alpha }q_{i}^{1-\alpha }\right]}^{\frac{1-\beta }{1-\alpha }}\right)-1}{\beta -1},$$

for all α>0, α≠1 and β≠1.

If we assume that h=id then (5) becomes Sharma–Mittal divergence.

When for all t>0, h(t)=log(te) then (5) becomes Rényi divergence of order α.

We substitute for $t={\left[\overset{n}{\underset{i=1}{\sum }}p_{i}^{\alpha }q_{i}^{1-\alpha }\right]}^{\frac{1-\beta }{1-\alpha }}$ and we have

$$h\left(t\right)=h\left({\left[\sum_{i=1}^{n}p_{i}^{\alpha }q_{i}^{1-\alpha }\right]}^{\frac{1-\beta }{1-\alpha }}\right)=\log\left({\left[\sum_{i=1}^{n}p_{i}^{\alpha }q_{i}^{1-\alpha }\right]}^{\frac{1-\beta }{1-\alpha }}e\right)=\log{\left[\sum_{i=1}^{n}p_{i}^{\alpha }q_{i}^{1-\alpha }\right]}^{\frac{1-\beta }{1-\alpha }}+1.$$

Hence, from (5)

$$SM_{h,\alpha ,\beta }\left(p,q\right)=\frac{\log{\left[\overset{n}{\underset{i=1}{\sum }}p_{i}^{\alpha }q_{i}^{1-\alpha }\right]}^{\frac{1-\beta }{1-\alpha }}}{\beta -1}=\frac{1}{\alpha -1}\log\sum_{i=1}^{n}p_{i}^{\alpha }q_{i}^{1-\alpha }=H_{\alpha }\left(p,q\right).$$

Let $\Psi :R_{+}\times R_{+}\to R$ be a differentiable function with respect to β and

$$\Psi \left(\alpha ,\beta \right)=h\left({\left[\sum_{i=1}^{n}p_{i}^{\alpha }q_{i}^{1-\alpha }\right]}^{\frac{1-\beta }{1-\alpha }}\right).$$

We assume that $h^{\prime }\left(1\right)=1$ and Ψ(α,1)=1. Then,

$$\lim_{\beta \to 1}\frac{\Psi (\alpha ,\beta )-\Psi (\alpha ,1)}{\beta -1}=\Psi^{\prime }\left(\alpha ,1\right)=h^{\prime }{\left({\left[\sum_{i=1}^{n}p_{i}^{\alpha }q_{i}^{1-\alpha }\right]}^{\frac{1-\beta }{1-\alpha }}\right)}_{|\beta =1}=$$

$${\frac{\partial }{\partial \beta }}_{{|}_{\beta =1}}h\left({\left[\sum_{i=1}^{n}p_{i}^{\alpha }q_{i}^{1-\alpha }\right]}^{\frac{1-\beta }{1-\alpha }}\right){\frac{\partial }{\partial \beta }}_{{|}_{\beta =1}}\left({\left[\sum_{i=1}^{n}p_{i}^{\alpha }q_{i}^{1-\alpha }\right]}^{\frac{1-\beta }{1-\alpha }}\right){\frac{\partial }{\partial \beta }}_{{|}_{\beta =1}}\left(\frac{1-\beta }{1-\alpha }\right)=$$

$$h^{\prime }\left(1\right){\left(\sum_{i=1}^{n}p_{i}^{\alpha }q_{i}^{1-\alpha }\right)}^{\frac{1-1}{1-\alpha }}\log\left(\sum_{i=1}^{n}p_{i}^{\alpha }q_{i}^{1-\alpha }\right)\left(\frac{-1}{1-\alpha }\right)=$$

$$h^{\prime }\left(1\right)\frac{1}{\alpha -1}\log\left(\sum_{i=1}^{n}p_{i}^{\alpha }q_{i}^{1-\alpha }\right)=\frac{1}{\alpha -1}\log\left(\sum_{i=1}^{n}p_{i}^{\alpha }q_{i}^{1-\alpha }\right).$$

Hence, the Sharma–Mittal h–divergence tends to Rényi divergence of order α.

**Remark** **1.** *If, additionally, α tends to 1 then based on the proof of the Equation (11) from [16], Sharma–Mittal h-divergence tends to relative entropy (called Kullback–Leibler divergence).*

Now we define a new *generalized (h,ϕ) Sharma–Mittal divergence* as follows

(6) $$SM_{h,\phi ,\alpha ,\beta }\left(p,q\right)=\frac{h\left({\left[\overset{n}{\underset{i=1}{\sum }}q_{i}\phi \left(\frac{p_{i}}{q_{i}},\alpha \right)\right]}^{\frac{1-\beta }{1-\alpha }}\right)-1}{\beta -1},$$

where $\phi :\left(0,+\infty \right)\times R_{+}\to R_{+}$ is an increasing, non-negative and differentiable function for β>1.

We assume that $F=\{f_{\alpha }:\left(0,\infty \right)\to R:\alpha \in R\}$ is a given family of functions such that $\overset{n}{\underset{i=1}{\sum }}q_{i}f_{\alpha_{{|}_{\alpha =1}}}\left(\frac{p_{i}}{q_{i}}\right)=1$ for α=1 and which are increasing, non-negative for α>1 and such that for every t∈(0,+∞) the function $\alpha ⟼f_{\alpha }\left(t\right)$ is differentiable.

According to [16] if we substitute the function $f_{\alpha }\left(\frac{p_{i}}{q_{i}}\right)$ from the family F for $\phi (\frac{p_{i}}{q_{i}},\alpha )$ then it stands that

$$\lim_{\beta \to 1}SM_{h,\phi ,\alpha ,\beta }\left(p,q\right)=R_{h,f_{\alpha }}\left(p,q\right).$$

We assume that h(1)=1. Then,

$$\lim_{\beta \to 1}SM_{h,\phi ,\alpha ,\beta }\left(p,q\right)=\lim_{\beta \to 1}\frac{h\left({\left[\overset{n}{\underset{i=1}{\sum }}q_{i}\phi \left(\frac{p_{i}}{q_{i}},\alpha \right)\right]}^{\frac{1-\beta }{1-\alpha }}\right)-1}{\beta -1}=$$

$$h^{\prime }\left({\left[\sum_{i=1}^{n}q_{i}\phi \left(\frac{p_{i}}{q_{i}},\alpha \right)\right]}^{0}\right)\cdot {\left[\sum_{i=1}^{n}q_{i}\phi \left(\frac{p_{i}}{q_{i}},\alpha \right)\right]}^{0}\left[\log\sum_{i=1}^{n}q_{i}\phi \left(\frac{p_{i}}{q_{i}},\alpha \right)\right]\frac{-1}{1-\alpha }=$$

$$h^{\prime }\left(1\right)\frac{1}{\alpha -1}\log\sum_{i=1}^{n}q_{i}\phi \left(\frac{p_{i}}{q_{i}},\alpha \right)=\frac{1}{\alpha -1}\log\sum_{i=1}^{n}q_{i}\phi \left(\frac{p_{i}}{q_{i}},\alpha \right)=$$

(7) $$\frac{1}{\alpha -1}\log\sum_{i=1}^{n}q_{i}f_{\alpha }\left(\frac{p_{i}}{q_{i}}\right)=R_{h,f_{\alpha }}\left(p,q\right).$$

**Remark** **2.** *If in (6) β→1 and $\phi \left(\frac{p_{i}}{q_{i}},\alpha \right)=f_{\alpha }\left(\frac{p_{i}}{q_{i}}\right)$ then the generalized (h,ϕ)–Sharma–Mittal divergence tends to generalized (h,F)–Rényi divergence.*

The function ϕ is the generalization of the function $f_{\alpha }\left(\frac{p_{i}}{q_{i}}\right)$ which is used for example in Csiszár *f*-divergence. Condition β→1 means that the limit of generalized Sharma–Mittal divergence is equal to generalized (h,F)–Rényi divergence. Hence we have implications for generalized forms of entropies.

**Remark** **3.** *Additionally, when in (6) α→1 and $\phi \left(\frac{p_{i}}{q_{i}},\alpha \right)={\left(\frac{p_{i}}{q_{i}}\right)}^{\alpha }$ then the generalized (h,ϕ)–Sharma–Mittal divergence tends to Kullback–Leibler divergence, because we have from Remark 2*

$$\lim_{\left(\alpha ,\beta )\to (1,1\right)}SM_{h,\phi ,\alpha ,\beta }\left(p,q\right)=\lim_{\alpha \to 1}\frac{\log\overset{n}{\underset{i=1}{\sum }}q_{i}\phi \left(\frac{p_{i}}{q_{i}},\alpha \right)-\log1}{\alpha -1}=\;{\frac{\partial }{\partial \alpha }}_{{|}_{\alpha =1}}\log\sum_{i=1}^{n}q_{i}{\left(\frac{p_{i}}{q_{i}}\right)}^{\alpha }=\frac{1}{\overset{n}{\underset{i=1}{\sum }}p_{i}}\sum_{i=1}^{n}q_{i}\frac{p_{i}}{q_{i}}\log\frac{p_{i}}{q_{i}}=\sum_{i=1}^{n}p_{i}\log\frac{p_{i}}{q_{i}}=H_{1}\left(p,q\right).$$

**Remark** **4.** *In (6), when the parameter β=α, the function h=id and $\phi \left(\frac{p_{i}}{q_{i}},\alpha \right)={\left(\frac{p_{i}}{q_{i}}\right)}^{\alpha }$ then the generalized (h,ϕ)–Sharma–Mittal divergence tends to the Tsallis f-divergence or order α.*

This work is more theoretical than practical. Therefore, the implications are formulated in the mathematical area that is from constructing general model which gives known specific cases.

## 3. Jensen–Sharma–Mittal and Jeffreys–Sharma–Mittal Divergences

The Jensen–Shannon divergence (Jensen–Shannon entropy) is defined as follows (see [17]):

$$\mathrm{Jen}\;\left(p,q\right)=\frac{1}{2}SM\left(p,\frac{p+q}{2}\right)+\frac{1}{2}SM\left(q,\frac{p+q}{2}\right).$$

The Jeffreys divergence (Jeffreys entropy) is defined as follows (see [17]):

Jef(p,q)=SM(p,q)+SM(q,p).

We introduce a new generalized (h,ϕ) Jensen–Sharma–Mittal divergence defined by

(8) $$\mathrm{Jen}\;SM_{\alpha ,\beta }^{h,\phi }\left(p,q\right)=\frac{1}{2}SM_{h,\phi ,\alpha ,\beta }\left(p,\frac{p+q}{2}\right)+\frac{1}{2}SM_{h,\phi ,\alpha ,\beta }\left(q,\frac{p+q}{2}\right)$$

with assumptions as before.

We similarly introduce a new generalized (h,ϕ) Jeffreys–Sharma–Mittal divergence as follows

(9) $$\mathrm{Jef}\;SM_{\alpha ,\beta }^{h,\phi }\left(p,q\right)=SM_{h,\phi ,\alpha ,\beta }\left(p,q\right)+SM_{h,\phi ,\alpha ,\beta }\left(q,p\right).$$

Taking into account inequality from [17]:

$$0\leq \mathrm{Jen}\;\left(p,q\right)\leq \frac{1}{2}\mathrm{Jef}\;\left(p,q\right),$$

describing the relation between the Jensen–Shannon and Jeffreys divergences, we could formulate the following:

(10) $$0\leq \mathrm{Jen}\;SM_{\alpha ,\beta }^{h,\phi }\left(p,q\right)\leq \frac{1}{2}\mathrm{Jef}\;SM_{\alpha ,\beta }^{h,\phi }\left(p,q\right).$$

We define the *Jensen–Sharma–Mittal h-divergence* where, in (8), $\phi \left(\frac{p_{i}}{q_{i}},\alpha \right)={\left(\frac{p_{i}}{q_{i}}\right)}^{\alpha }$. Then, it takes the form:

(11) $$\mathrm{Jen}\;SM_{\alpha ,\beta }^{h}\left(p,q\right)=\frac{1}{2}SM_{h,\alpha ,\beta }\left(p,\frac{p+q}{2}\right)+\frac{1}{2}SM_{h,\alpha ,\beta }\left(q,\frac{p+q}{2}\right)$$

In the same way, we define the *Jeffreys–Sharma–Mittal h–divergence*:

(12) $$\mathrm{Jef}\;SM_{\alpha ,\beta }^{h}\left(p,q\right)=SM_{h,\alpha ,\beta }\left(p,q\right)+SM_{h,\alpha ,\beta }\left(q,p\right).$$

Additionally, if the function h(t)=t then we define the Jensen–Sharma–Mittal and the Jeffreys–Sharma–Mittal divergences of order α and degree β, respectively.

(13) $$\mathrm{Jen}\;SM_{\alpha ,\beta }\left(p,q\right)=\frac{1}{2}SM_{\alpha ,\beta }\left(p,\frac{p+q}{2}\right)+\frac{1}{2}SM_{\alpha ,\beta }\left(q,\frac{p+q}{2}\right),$$

(14) $$\mathrm{Jef}\;SM_{\alpha ,\beta }\left(p,q\right)=SM_{\alpha ,\beta }\left(p,q\right)+SM_{\alpha ,\beta }\left(q,p\right).$$

When in (8) and (9) β→1 and we substitute for $\phi \left(\frac{p_{i}}{q_{i}},\alpha \right)=f_{\alpha }\left(\frac{p_{i}}{q_{i}}\right)$ then we obtain, defined in [16], the generalized (h,F) Jensen–Rényi and Jeffreys–Rényi divergences, respectively:

$$\mathrm{Jen}\;SM_{\alpha ,1}^{h,f_{\alpha }}\left(p,q\right)=\mathrm{Jen}\;R_{h,f_{\alpha }}\left(p,q\right)=\frac{1}{2}R_{h,f_{\alpha }}\left(p,\frac{p+q}{2}\right)+\frac{1}{2}R_{h,f_{\alpha }}\left(q,\frac{p+q}{2}\right),$$

(15) $$\mathrm{Jef}\;SM_{\alpha ,1}^{h,f_{\alpha }}\left(p,q\right)=\mathrm{Jef}\;R_{h,f_{\alpha }}\left(p,q\right)=R_{h,f_{\alpha }}\left(p,q\right)+R_{h,f_{\alpha }}\left(q,p\right).$$

The following theorem is the generalization and refinement of the inequalities for some known divergences and provides lower and upper bounds for the generalized (h,ϕ) Jeffreys–Sharma–Mittal divergence in order to a more accurate estimation of its uncertainty measure.

**Theorem** **1.** *Let $p=(p_{1},\ldots ,p_{n})$ and $q=(q_{1},\ldots ,q_{n})$ be two discrete probability distributions with $p_{i}>0$, $q_{i}>0$, $\frac{q_{i}}{p_{i}}\in I_{0}$, $\frac{p_{i}}{q_{i}}\in I_{0}$, i=1,…,n, where $I_{0}\subset R$ is an interval, such that $1\in I_{0}$. Let $\phi :I_{0}\times R_{+}\to R_{+}$ be an increasing, non-negative and differentiable function for which $\overset{n}{\underset{i=1}{\sum }}q_{i}\phi \left(\frac{p_{i}}{q_{i}},\alpha \right)\geq 1$ and $\overset{n}{\underset{i=1}{\sum }}p_{i}\phi \left(\frac{q_{i}}{p_{i}},\alpha \right)\geq 1$ where 1<β≤α, $\alpha \in R_{+}\setminus \left\{1\right\}$ and $h:I_{0}\to R$ be a convex and increasing function on $I_{0}$.*

*Then, the following inequalities are valid:*

$$\frac{1}{\alpha -1}\log\prod_{i=1}^{n}{\left(\phi \left(\frac{p_{i}}{q_{i}},\alpha \right)\right)}^{q_{i}}{\left(\phi \left(\frac{q_{i}}{p_{i}},\alpha \right)\right)}^{p_{i}}\leq \mathrm{Jef}\;SM_{\alpha ,\beta }^{h,\phi }\left(p,q\right)\leq$$

(16) $$\frac{1}{\beta -1}\left(\mathrm{Jef}\;C_{h\circ \phi }\left(p,q\right)-2\right).$$

**Proof.**  Taking into account the assumptions, we could formulate the following inequality:

(17) $${\left[\sum_{i=1}^{n}q_{i}\phi \left(\frac{p_{i}}{q_{i}},\alpha \right)\right]}^{\frac{1-\beta }{1-\alpha }}\leq \sum_{i=1}^{n}q_{i}\phi \left(\frac{p_{i}}{q_{i}},\alpha \right).$$

The function *h* is increasing and convex, therefore, from (4) and (17) we obtain inequalities:

(18) $$h\left({\left[\sum_{i=1}^{n}q_{i}\phi \left(\frac{p_{i}}{q_{i}},\alpha \right)\right]}^{\frac{1-\beta }{1-\alpha }}\right)\leq h\left(\sum_{i=1}^{n}q_{i}\phi \left(\frac{p_{i}}{q_{i}},\alpha \right)\right)\leq \sum_{i=1}^{n}q_{i}\left(h\circ \phi \right)\left(\frac{p_{i}}{q_{i}},\alpha \right).$$

In the same way, we obtain the following inequalities:

(19) $$h\left({\left[\sum_{i=1}^{n}p_{i}\phi \left(\frac{q_{i}}{p_{i}},\alpha \right)\right]}^{\frac{1-\beta }{1-\alpha }}\right)\leq h\left(\sum_{i=1}^{n}p_{i}\phi \left(\frac{q_{i}}{p_{i}},\alpha \right)\right)\leq \sum_{i=1}^{n}p_{i}\left(h\circ \phi \right)\left(\frac{q_{i}}{p_{i}},\alpha \right).$$

From (9) we have

$$\mathrm{Jef}\;SM_{\alpha ,\beta }^{h,\phi }\left(p,q\right)=SM_{h,\phi ,\alpha ,\beta }\left(p,q\right)+SM_{h,\phi ,\alpha ,\beta }\left(q,p\right)=$$

$$\frac{h\left({\left[\overset{n}{\underset{i=1}{\sum }}q_{i}\phi \left(\frac{p_{i}}{q_{i}},\alpha \right)\right]}^{\frac{1-\beta }{1-\alpha }}\right)-1}{\beta -1}+\frac{h\left({\left[\overset{n}{\underset{i=1}{\sum }}p_{i}\phi \left(\frac{q_{i}}{p_{i}},\alpha \right)\right]}^{\frac{1-\beta }{1-\alpha }}\right)-1}{\beta -1}$$

Taking into account (2), (18), (19) and the definition of Jeffreys divergence, it stands that:

$$\mathrm{Jef}\;SM_{\alpha ,\beta }^{h,\phi }\left(p,q\right)\leq \frac{\overset{n}{\underset{i=1}{\sum }}q_{i}\left(h\circ \phi \right)\left(\frac{p_{i}}{q_{i}},\alpha \right)-1}{\beta -1}+\frac{\overset{n}{\underset{i=1}{\sum }}p_{i}\left(h\circ \phi \right)\left(\frac{q_{i}}{p_{i}},\alpha \right)-1}{\beta -1}=$$

(20) $$\frac{\overset{n}{\underset{i=1}{\sum }}q_{i}\left(h\circ \phi \right)\left(\frac{p_{i}}{q_{i}},\alpha \right)+\overset{n}{\underset{i=1}{\sum }}p_{i}\left(h\circ \phi \right)\left(\frac{q_{i}}{p_{i}},\alpha \right)-2}{\beta -1}=\frac{\mathrm{Jef}\;C_{h\circ \phi }\left(p,q\right)-2}{\beta -1}.$$

The above inequality is the upper bound for generalized (h,ϕ) Jeffreys–Sharma–Mittal divergence.

By using the convexity of the function *h* with h(1)=1 the following inequality is valid for β>1:

(21) $$\frac{h\left({\left[\overset{n}{\underset{i=1}{\sum }}q_{i}\phi \left(\frac{p_{i}}{q_{i}},\alpha \right)\right]}^{\frac{1-\beta }{1-\alpha }}\right)-1}{\beta -1}\geq {\frac{\partial }{\partial \beta }}_{{|}_{\beta =1}}h\left({\left[\sum_{i=1}^{n}q_{i}\phi \left(\frac{p_{i}}{q_{i}},\alpha \right)\right]}^{\frac{1-\beta }{1-\alpha }}\right).$$

From (7) the above derivative function is equal to: $\frac{1}{\alpha -1}\log\sum_{i=1}^{n}q_{i}\phi \left(\frac{p_{i}}{q_{i}},\alpha \right)$.

The function f(t)=logt is concave and increasing. Then, it stands that:

(22) $$\frac{1}{\alpha -1}\log\sum_{i=1}^{n}q_{i}\phi \left(\frac{p_{i}}{q_{i}},\alpha \right)\geq \frac{1}{\alpha -1}\sum_{i=1}^{n}q_{i}\log\left(\phi \left(\frac{p_{i}}{q_{i}},\alpha \right)\right).$$

Hence, from (21) and (22) we have the inequality:

(23) $$\frac{h\left({\left[\overset{n}{\underset{i=1}{\sum }}q_{i}\phi \left(\frac{p_{i}}{q_{i}},\alpha \right)\right]}^{\frac{1-\beta }{1-\alpha }}\right)-1}{\beta -1}\geq \frac{1}{\alpha -1}\sum_{i=1}^{n}q_{i}\log\left(\phi \left(\frac{p_{i}}{q_{i}},\alpha \right)\right).$$

Similarly, we obtain the second inequality:

(24) $$\frac{h\left({\left[\overset{n}{\underset{i=1}{\sum }}p_{i}\phi \left(\frac{q_{i}}{p_{i}},\alpha \right)\right]}^{\frac{1-\beta }{1-\alpha }}\right)-1}{\beta -1}\geq \frac{1}{\alpha -1}\sum_{i=1}^{n}p_{i}\log\left(\phi \left(\frac{q_{i}}{p_{i}},\alpha \right)\right).$$

We have from (6), (23) and (24) that:

$$SM_{h,\phi ,\alpha ,\beta }\left(p,q\right)\geq \frac{1}{\alpha -1}\sum_{i=1}^{n}q_{i}\log\left(\phi \left(\frac{p_{i}}{q_{i}},\alpha \right)\right),$$

$$SM_{h,\phi ,\alpha ,\beta }\left(q,p\right)\geq \frac{1}{\alpha -1}\sum_{i=1}^{n}p_{i}\log\left(\phi \left(\frac{q_{i}}{p_{i}},\alpha \right)\right).$$

Then, by using the definition (9) we have:

$$\mathrm{Jef}\;SM_{\alpha ,\beta }^{h,\phi }\left(p,q\right)\geq \frac{1}{\alpha -1}\left(\log\prod_{i=1}^{n}{\left(\phi \left(\frac{p_{i}}{q_{i}},\alpha \right)\right)}^{q_{i}}+\log\prod_{i=1}^{n}{\left(\phi \left(\frac{q_{i}}{p_{i}},\alpha \right)\right)}^{p_{i}}\right)=$$

(25) $$\frac{1}{\alpha -1}\log\prod_{i=1}^{n}{\left(\phi \left(\frac{p_{i}}{q_{i}},\alpha \right)\right)}^{q_{i}}{\left(\phi \left(\frac{q_{i}}{p_{i}},\alpha \right)\right)}^{p_{i}}.$$

This result is the lower bound of the generalized (h,ϕ) Jeffreys–Sharma–Mittal divergence.

Combining (20) and (25) we obtain the expected inequalities (16). □

**Corollary** **1.** *When we substitute for $\phi \left(\frac{p_{i}}{q_{i}},\alpha \right)={\left(\frac{p_{i}}{q_{i}}\right)}^{\alpha }$ then from (16) we obtain the inequalities for Jeffreys–Sharma–Mittal h-divergence:*

$$\frac{1}{\alpha -1}\log\prod_{i=1}^{n}{\left(\frac{p_{i}}{q_{i}}\right)}^{\alpha (q_{i}-p_{i})}\leq \mathrm{Jef}\;SM_{\alpha ,\beta }^{h}\left(p,q\right)\leq$$

$$\frac{\overset{n}{\underset{i=1}{\sum }}q_{i}h\left({\left(\frac{p_{i}}{q_{i}}\right)}^{\alpha }\right)+\overset{n}{\underset{i=1}{\sum }}p_{i}h\left({\left(\frac{q_{i}}{p_{i}}\right)}^{\alpha }\right)-2}{\beta -1}.$$

We now formulate the theorem thanks to which the estimation of the generalized (h,ϕ) Jensen–Sharma–Mittal divergence will be possible.

**Theorem** **2.** *Let $p=(p_{1},\ldots ,p_{n})$ and $q=(q_{1},\ldots ,q_{n})$ be two discrete probability distributions with $p_{i}>0$, $q_{i}>0$, $\frac{q_{i}}{p_{i}}\in I_{0}$, $\frac{p_{i}}{q_{i}}\in I_{0}$, i=1,…,n, where $I_{0}\subset R$ is an interval such that $1\in I_{0}$. Let $\phi :I_{0}\times R_{+}\to R_{+}$ be an increasing, non-negative and differentiable function for which $\overset{n}{\underset{i=1}{\sum }}\frac{p_{i}+q_{i}}{2}\phi \left(\frac{2p_{i}}{p_{i}+q_{i}},\alpha \right)\geq 1$ and $\overset{n}{\underset{i=1}{\sum }}\frac{p_{i}+q_{i}}{2}\phi \left(\frac{2q_{i}}{p_{i}+q_{i}},\alpha \right)\geq 1$ where 1<β≤α, $\alpha \in R_{+}\setminus \left\{1\right\}$ and $h:I_{0}\to R$ be a convex and increasing function on $I_{0}$.*

*Then, the following inequalities are valid:*

$$\frac{1}{2(\alpha -1)}\log\prod_{i=1}^{n}{\left(\phi \left(\frac{2p_{i}}{p_{i}+q_{i}},\alpha \right)\phi \left(\frac{2q_{i}}{p_{i}+q_{i}},\alpha \right)\right)}^{\frac{p_{i}+q_{i}}{2}}\leq \mathrm{Jen}\;SM_{\alpha ,\beta }^{h,\phi }\left(p,q\right)\leq$$

(26) $$\frac{\overset{n}{\underset{i=1}{\sum }}\left(p_{i}+q_{i}\right)\left[\left(h\circ \phi \right)\left(\frac{2p_{i}}{p_{i}+q_{i}},\alpha \right)+\left(h\circ \phi \right)\left(\frac{2q_{i}}{p_{i}+q_{i}},\alpha \right)\right]-4}{4(\beta -1)}$$

**Proof.**  Let’s consider the function

(27) $$\frac{h\left({\left[\overset{n}{\underset{i=1}{\sum }}\frac{p_{i}+q_{i}}{2}\phi \left(\frac{p_{i}}{\frac{p_{i}+q_{i}}{2}},\alpha \right)\right]}^{\frac{1-\beta }{1-\alpha }}\right)-1}{\beta -1}.$$

Using the assumptions that the function *h* is differentiable, convex and h(1)=1 we could formulate the following inequality:

(28) $$\frac{h\left({\left[\overset{n}{\underset{i=1}{\sum }}\frac{p_{i}+q_{i}}{2}\phi \left(\frac{p_{i}}{\frac{p_{i}+q_{i}}{2}},\alpha \right)\right]}^{\frac{1-\beta }{1-\alpha }}\right)-1}{\beta -1}\geq {\frac{\partial }{\partial \beta }}_{{|}_{\beta =1}}h\left({\left[\sum_{i=1}^{n}\frac{p_{i}+q_{i}}{2}\phi \left(\frac{p_{i}}{\frac{p_{i}+q_{i}}{2}},\alpha \right)\right]}^{\frac{1-\beta }{1-\alpha }}\right).$$

Then, (28) is equal to:

$$\left[\log\sum_{i=1}^{n}\frac{p_{i}+q_{i}}{2}\phi \left(\frac{p_{i}}{\frac{p_{i}+q_{i}}{2}},\alpha \right)\right]\left(\frac{-1}{1-\alpha }\right)=\frac{1}{\alpha -1}\log\sum_{i=1}^{n}\frac{p_{i}+q_{i}}{2}\phi \left(\frac{p_{i}}{\frac{p_{i}+q_{i}}{2}},\alpha \right).$$

Taking into account concavity of the function log, we have that:

$$\frac{1}{\alpha -1}\log\sum_{i=1}^{n}\frac{p_{i}+q_{i}}{2}\phi \left(\frac{p_{i}}{\frac{p_{i}+q_{i}}{2}},\alpha \right)\geq \frac{1}{\alpha -1}\sum_{i=1}^{n}\frac{p_{i}+q_{i}}{2}\log\left(\phi \left(\frac{p_{i}}{\frac{p_{i}+q_{i}}{2}},\alpha \right)\right).$$

Then, we obtain that (27) is greater than

(29) $$\frac{1}{\alpha -1}\log\prod_{i=1}^{n}{\left(\phi \left(\frac{p_{i}}{\frac{p_{i}+q_{i}}{2}},\alpha \right)\right)}^{\frac{p_{i}+q_{i}}{2}}.$$

We do the same with the function

(30) $$\frac{h\left({\left[\overset{n}{\underset{i=1}{\sum }}\frac{p_{i}+q_{i}}{2}\phi \left(\frac{q_{i}}{\frac{p_{i}+q_{i}}{2}},\alpha \right)\right]}^{\frac{1-\beta }{1-\alpha }}\right)-1}{\beta -1}.$$

Hence, we have that (30) is greater than

(31) $$\frac{1}{\alpha -1}\log\prod_{i=1}^{n}{\left(\phi \left(\frac{q_{i}}{\frac{p_{i}+q_{i}}{2}},\alpha \right)\right)}^{\frac{p_{i}+q_{i}}{2}}.$$

Then, combining (27), (29)–(31), and using the definition (8) the following inequality occurs

(32) $$\mathrm{Jen}\;SM_{\alpha ,\beta }^{h,\phi }\left(p,q\right)\geq \frac{1}{2(\alpha -1)}\log\prod_{i=1}^{n}{\left(\phi \left(\frac{2p_{i}}{p_{i}+q_{i}},\alpha \right)\phi \left(\frac{2q_{i}}{p_{i}+q_{i}},\alpha \right)\right)}^{\frac{p_{i}+q_{i}}{2}}$$

and it is the lower bound of the generalized (h,ϕ) Jensen–Sharma–Mittal divergence.

When we consider the function

(33) $$h\left({\left[\sum_{i=1}^{n}\frac{p_{i}+q_{i}}{2}\phi \left(\frac{p_{i}}{\frac{p_{i}+q_{i}}{2}},\alpha \right)\right]}^{\frac{1-\beta }{1-\alpha }}\right)$$

with 1<β≤α then for the convex and increasing function *h* we have from (4) that (33) is smaller than

(34) $$h\left(\sum_{i=1}^{n}\frac{p_{i}+q_{i}}{2}\phi \left(\frac{p_{i}}{\frac{p_{i}+q_{i}}{2}},\alpha \right)\right)\leq \sum_{i=1}^{n}\frac{p_{i}+q_{i}}{2}\left(h\circ \phi \right)\left(\frac{p_{i}}{\frac{p_{i}+q_{i}}{2}},\alpha \right).$$

In a similar way we conclude the following inequality for the function

(35) $$h\left({\left[\sum_{i=1}^{n}\frac{p_{i}+q_{i}}{2}\phi \left(\frac{q_{i}}{\frac{p_{i}+q_{i}}{2}},\alpha \right)\right]}^{\frac{1-\beta }{1-\alpha }}\right)$$

and we have

(36) $$h\left(\sum_{i=1}^{n}\frac{p_{i}+q_{i}}{2}\phi \left(\frac{q_{i}}{\frac{p_{i}+q_{i}}{2}},\alpha \right)\right)\leq \sum_{i=1}^{n}\frac{p_{i}+q_{i}}{2}\left(h\circ \phi \right)\left(\frac{q_{i}}{\frac{p_{i}+q_{i}}{2}},\alpha \right).$$

Then combining (33)–(36) and the definition (8) with the proper transformations we obtain the inequality

(37) $$\mathrm{Jen}\;SM_{\alpha ,\beta }^{h,\phi }\left(p,q\right)\leq \frac{\overset{n}{\underset{i=1}{\sum }}\left(p_{i}+q_{i}\right)\left[\left(h\circ \phi \right)\left(\frac{2p_{i}}{p_{i}+q_{i}},\alpha \right)+\left(h\circ \phi \right)\left(\frac{2q_{i}}{p_{i}+q_{i}},\alpha \right)\right]-4}{4(\beta -1)}$$

which is the upper bound of the generalized (h,ϕ) Jensen–Sharma–Mittal divergence.

When we take into account (32) and (37), then we obtain (26). □

**Corollary** **2.** *When we substitute for $\phi \left(\frac{2p_{i}}{p_{i}+q_{i}},\alpha \right)={\left(\frac{2p_{i}}{p_{i}+q_{i}}\right)}^{\alpha }$ and for $\phi \left(\frac{2q_{i}}{p_{i}+q_{i}},\alpha \right)={\left(\frac{2q_{i}}{p_{i}+q_{i}}\right)}^{\alpha }$ then from (26) we obtain the inequalities for Jensen–Sharma–Mittal h–divergence:*

$$\frac{1}{2(\alpha -1)}\log\prod_{i=1}^{n}{\left[\frac{4p_{i}q_{i}}{{\left(p_{i}+q_{i}\right)}^{2}}\right]}^{\frac{\alpha (p_{i}+q_{i})}{2}}\leq \mathrm{Jen}\;SM_{\alpha ,\beta }^{h}\left(p,q\right)\leq$$

$$\frac{\overset{n}{\underset{i=1}{\sum }}\left(p_{i}+q_{i}\right)\left[h\left({\left(\frac{2p_{i}}{p_{i}+q_{i}}\right)}^{\alpha }\right)+h\left({\left(\frac{2q_{i}}{p_{i}+q_{i}}\right)}^{\alpha }\right)\right]-4}{4(\beta -1)}.$$

**Remark** **5.** *It could be seen that the lower bounds for both Jeffreys (25) and Jensen (32) Sharma–Mittal (h,ϕ) divergences are independent of the function h.*

**Remark** **6.** *Taking into account the inequality (10) we obtain the alternative upper bound for the Jensen–Sharma–Mittal and the lower bound for the Jeffreys–Sharma–Mittal generalized (h,ϕ) divergences, respectively.*

$$\mathrm{Jen}\;SM_{\alpha ,\beta }^{h,\phi }\left(p,q\right)\leq \frac{h\left({\left[\overset{n}{\underset{i=1}{\sum }}q_{i}\phi \left(\frac{p_{i}}{q_{i}},\alpha \right)\right]}^{\frac{1-\beta }{1-\alpha }}\right)+h\left({\left[\overset{n}{\underset{i=1}{\sum }}q_{i}\phi \left(\frac{p_{i}}{q_{i}},\alpha \right)\right]}^{\frac{1-\beta }{1-\alpha }}\right)-2}{2(\beta -1)},$$

$$\mathrm{Jef}\;SM_{\alpha ,\beta }^{h,\phi }\left(p,q\right)\geq \frac{h\left({\left[\overset{n}{\underset{i=1}{\sum }}\frac{p_{i}+q_{i}}{2}\phi \left(\frac{2p_{i}}{p_{i}+q_{i}},\alpha \right)\right]}^{\frac{1-\beta }{1-\alpha }}\right)-1}{\beta -1}+$$

$$\frac{h\left({\left[\overset{n}{\underset{i=1}{\sum }}\frac{p_{i}+q_{i}}{2}\phi \left(\frac{2q_{i}}{p_{i}+q_{i}},\alpha \right)\right]}^{\frac{1-\beta }{1-\alpha }}\right)-1}{\beta -1}.$$

## 4. Applications

In this section we show how our theory works.

### 4.1. Bounds for Sharma–Mittal Divergences

For the functions h(t)=t, $\phi \left(t,\alpha \right)=t^{\alpha }$ and based on Theorems 1 and 3 we obtain the lower and upper bounds for Jeffreys–Sharma–Mittal and Jensen–Sharma–Mittal divergences, respectively, as follows

(38) $$\frac{1}{\alpha -1}\log\prod_{i=1}^{n}{\left(\frac{p_{i}}{q_{i}}\right)}^{\alpha (q_{i}-p_{i})}\leq \mathrm{Jef}\;SM_{\alpha ,\beta }\left(p,q\right)\leq \frac{\overset{n}{\underset{i=1}{\sum }}{\left(p_{i}q_{i}\right)}^{\alpha }\left(q_{i}^{1-2\alpha }+p_{i}^{1-2\alpha }\right)-2}{\beta -1}$$

(39) $$\frac{1}{2(\alpha -1)}\log\prod_{i=1}^{n}{\left(\frac{2\sqrt{p_{i}q_{i}}}{p_{i}+q_{i}}\right)}^{\alpha (p_{i}+q_{i})}\leq \mathrm{Jen}\;SM_{\alpha ,\beta }\left(p,q\right)\leq \frac{\overset{n}{\underset{i=1}{\sum }}{\left(\frac{p_{i}+q_{i}}{2}\right)}^{1-\alpha }\left(p_{i}^{\alpha }+q_{i}^{\alpha }\right)-2}{2(\beta -1)}.$$

**Remark** **7.** *The above lower bounds (38) and (39) are the same for Rényi types divergences because they are independent of the parameter β which in that case approaches 1.*

**Remark** **8.** *Substituting different values for the parameters α, β, such that 1<β≤α and taking into account the assumptions from the Theorems 1 and 3 about the functions h and ϕ we could formulate new types of divergences and related inequalities which are based on the generalized (h,ϕ) Sharma–Mittal divergence.*

### 4.2. Bounds for Tsallis Divergences

When we make the same assumptions as for Sharma–Mittal divergences with additional that β=α we obtain the bounds for Tsallis type divergences as follows

$$\frac{1}{\alpha -1}\log\prod_{i=1}^{n}{\left(\frac{p_{i}}{q_{i}}\right)}^{\alpha (q_{i}-p_{i})}\leq \mathrm{Jef}\;T_{\alpha }\left(p,q\right)\leq \frac{\overset{n}{\underset{i=1}{\sum }}{\left(p_{i}q_{i}\right)}^{\alpha }\left(q_{i}^{1-2\alpha }+p_{i}^{1-2\alpha }\right)-2}{\alpha -1}$$

$$\frac{1}{2(\alpha -1)}\log\prod_{i=1}^{n}{\left(\frac{2\sqrt{p_{i}q_{i}}}{p_{i}+q_{i}}\right)}^{\alpha (p_{i}+q_{i})}\leq \mathrm{Jen}\;T_{\alpha }\left(p,q\right)\leq \frac{\overset{n}{\underset{i=1}{\sum }}{\left(\frac{p_{i}+q_{i}}{2}\right)}^{1-\alpha }\left(p_{i}^{\alpha }+q_{i}^{\alpha }\right)-2}{2(\alpha -1)}.$$

### 4.3. Bounds for Kullback–Leibler Divergences

When we have the same situation as in case of Tsallis divergence that is h(t)=t, $\phi \left(t,\alpha \right)=t^{\alpha }$, α=β and additionally both α and β approach 1 then we obtain new upper bounds for Jeffreys and Jensen–Shannon divergences, respectively.

$$\mathrm{Jef}\;S\left(p,q\right)\leq \sum_{i=1}^{n}\left(p_{i}+q_{i}\right)\log p_{i}q_{i},$$

$$\mathrm{Jen}\;S\left(p,q\right)\leq \sum_{i=1}^{n}\left[p_{i}\log p_{i}+q_{i}\log q_{i}-\left(p_{i}+q_{i}\right)\log\left(p_{i}+q_{i}\right)\right].$$

The last inequality is equivalent to JenS(p,q)≥2log2.

## 5. Summary

In this paper, new types of entropy have been defined, which are generalizations of others known and used so far in information theory.

The manuscript deals more with issues in the field of pure mathematics, therefore the standard axioms of entropy used in thermodynamics could, in this case, be extended by other assumptions and properties.

These divergences have been introduced for new physical interpretations which could be generated.

Generalized Sharma–Mittal and consequently Jensen–Sharma–Mittal and Jeffrey–Sharma–Mittal divergences have been defined for obtaining better estimates for known entropies, which will allow to more accurately determination of the dispersion measure of different distributions.

The derived inequalities have both upper and lower limits for the considered f-divergences. As a consequence, we obtain specific estimates for some new order measures. Hence they provide much wider interpretation possibilities in comparing probability distributions in the sense of mutual distances in different spaces.

In the era of advancing quantum mechanics, scientists are striving to build a quantum computer with very high computing power. The obtained results, despite their mathematical and analytical complexity, will very quickly generate specific numerical intervals which are an estimation of new introduced entropies. Therefore, such results as in this paper will be very useful in developing information theory issues.

This work is from the area of pure mathematics, therefore it is more theoretical than practical and makes it possible to find the existing known entropies by means of new defined generalizations. These generalizations can be used for interpreting various physical phenomena. The aim of this manuscript was to provide some new theoretical solutions for physicists who, with their knowledge and experience, will be able to look for new applications.

## Data Availability

Not applicable.
